# Direct Pulp Capping of Dental Pulp with Two Different Autologous Platelet Concentrates A-PRF+ and H-PRF—A Report on Two Cases

**DOI:** 10.3390/dj14010048

**Published:** 2026-01-12

**Authors:** Janet Kirilova, Dimitar Yovchev

**Affiliations:** 1 Department of Conservative Dentistry, Faculty of Dental Medicine, Medical University, 1000 Sofia, Bulgaria; 2 Department of Imaging and Oral Diagnostics, Faculty of Dental Medicine, Medical University, 1000 Sofia, Bulgaria; d.yovchev@fdm.mu-sofia.bg

**Keywords:** reversible pulpitis, direct pulp capping, platelet-rich fibrin, A-PRF+, H-PRF, dentin bridge, autologous platelet concentrates

## Abstract

**Background**: Autologous platelet concentrates, including platelet-rich fibrin (PRF) matrices, have been proposed as biologically active scaffolds for vital pulp therapy. Evidence on the clinical use of different solid PRF matrices for direct pulp capping remains limited. **Objective**: The aim of this study is to describe and monitor two clinical cases of reversible pulpitis treated with direct pulp capping using two PRF membranes prepared by different centrifugation approaches, namely advanced platelet-rich fibrin plus (A-PRF+) and horizontal platelet-rich fibrin plus (H-PRF). **Methods**: In Case 1, A-PRF+ was prepared using a fixed-angle centrifugation protocol; in Case 2, H-PRF was prepared using a horizontal centrifugation protocol. In both cases, deep carious lesions with small carious pulp exposures (<1.5 mm) were managed by caries removal, ozone-assisted dentin disinfection, and direct pulp capping with the respective PRF membrane, followed by temporary calcium-silicate cement definitive coronal restoration. Clinical and radiographic follow-up, including cone-beam computed tomography, was performed for up to 12 months. **Results**: In Case 1 (A-PRF+), reparative dentin bridge formation was confirmed at 90 days, with a thickness of 0.2 mm. In Case 2 (H-PRF), reparative dentin was observed within 46 days, with a thickness of 0.28 mm. In both cases, pulp vitality was maintained, and no clinical symptoms or periapical changes were detected during the 12-month follow-up. **Conclusions**: These two cases suggest that direct pulp capping using PRF membranes (A-PRF+ or H-PRF), combined with ozone-assisted dentin disinfection and adequate coronal sealing, may be associated with maintained pulp vitality and hard-tissue repair after carious pulp exposure diagnosed as reversible pulpitis. Due to the descriptive two-case design and major confounding factors (including age and lesion characteristics), no comparative conclusions can be drawn. Prospective controlled clinical studies with standardized protocols are warranted.

## 1. Introduction

Autologous platelet concentrates (APCs) are widely applied in dental medicine as biologically active derived from a patient’s own blood. These products are also referred to by various terms, including blood concentrates, platelet concentrates, autologous blood biomaterials, and autologous platelet concentrates [1,2]. In this article, we will use the term autologous platelet concentrates. First-generation autologous platelet concentrates include platelet-rich plasma (PRP), whereas second-generation products comprise platelet-rich fibrin (PRF) [3]. PRP requires anticoagulants and, in some protocols, bovine thrombin, and it is characterized by a rapid, almost immediate burst release of growth factors, which are not evenly distributed within the plasma. In contrast, in PRF, the growth factors are trapped within a three-dimensional fibrin matrix, are more homogeneously distributed throughout the fibrin network, and are released gradually over a longer period (up to approximately two weeks). This pattern implies a continuous, slow release of growth factors and a more sustained stimulation of wound-healing processes. These features have led to the development and adoption of PRF as an anticoagulant-free, more physiological, and clinically convenient alternative [3]. The biological properties of autologous platelet concentrates make them suitable for regenerative endodontics, including vital pulp therapy, managing chronic apical periodontitis, and treating immature teeth with incomplete root development [4].

PRF is obtained by centrifuging autologous blood. Centrifugation separates and removes erythrocytes, leaving plasma, platelets, leukocytes, and growth factors and cytokines released from platelets, which together support wound healing [3]. In second-generation autologous platelet concentrates, a three-dimensional fibrin network (matrix or scaffold) is formed, which provides structural support for cell migration and tissue regeneration [5,6,7]. Platelet-derived growth factors stimulate cell proliferation and differentiation, while leukocytes secrete signaling molecules that promote tissue repair and the recruitment of mesenchymal stem cells [8]. Thus, covering a wound such as pulp exposure or periapical tissue damage with PRF can accelerate healing and help restore normal tooth function, representing a form of bioengineering of the affected tissues [9,10,11].

The preparation of PRF depends on the centrifuge design and the specific protocol used [9,11]. Two main centrifuge types are employed in clinical practice, which are vertical-type centrifuges with a fixed-angle rotor and horizontal-type centrifuges. The orientation/angulation of centrifugation—fixed-angle vs. horizontal—is particularly important, as it influences the sedimentation behavior of cellular components, the distribution of platelets and leukocytes, and the density and architecture of the final fibrin matrix [11,12,13]. In addition, the blood collection tube characteristics (material and surface treatment) significantly affect clot formation and the quality of the resulting biomaterial [5,9].

In vertical-type centrifuges, modified low-speed protocols have been introduced to enhance PRF’s biological properties. Advanced platelet-rich fibrin plus (A-PRF+) is solid leukocyte- and platelet-rich fibrin membrane obtained using a modified low-speed centrifugation protocol in fixed-angle centrifuges. It is primarily used as a membrane or in combination with bone graft materials to form so-called “sticky bone” [6]. A-PRF+ is a leukocyte- and platelet-rich fibrin biomaterial with a porous fibrin architecture that allows for a gradual release of growth factors and cytokines, supporting angiogenesis, cell proliferation, and tissue regeneration [5,6,7,8].

Horizontal-type centrifuge was developed to further optimize cell capture and fibrin organization. Horizontal platelet-rich fibrin (H-PRF) protocols promote a more uniform distribution of platelets and leukocytes throughout the fibrin matrix compared with fixed-angle centrifugation systems, resulting in a dense and homogeneous fibrin network [11,12]. H-PRF is a dense fibrin clot produced using horizontal-type centrifugation without anticoagulants. It can be used as a membrane, incorporated into “sticky bone”, depending on the protocol [11,12]. Laboratory studies have demonstrated that PRF prepared using horizontal centrifugation contains approximately 3.5 times platelet and leukocyte yields compared with PRF obtained using conventional fixed-angle centrifugation, reflecting improved cell capture rather than stratification into a distinct buffy coat layer [11,12,13].

Clinical and experimental evidence supports the use of PRF in regenerative endodontics. In chronic apical periodontitis, the application of A-PRF+ has been associated with periapical tissues healing faster compared with treatment without PRF [14]. In immature permanent teeth with necrotic pulps, the use of PRF has yielded approximately 88% clinical success [15]. Both studies are randomized clinical trials. For direct pulp capping in teeth with reversible pulpitis, a randomized clinical trial found no statistically significant difference between PRF and MTA groups regarding dentin bridge thickness, suggesting that platelet concentrates may represent a viable option for vital pulp therapy in adult permanent teeth [16]. Clinical follow-up of cases treated with A-PRF+ has shown a tendency toward rapid dentin bridge formation within 3–5 months [17].

In vitro data further underline the potential of different PRF formulations. A study using human dental pulp stem cells (DPSCs) demonstrated that A-PRF+, Gel A-PRF+, and H-PRF significantly stimulate DPSC migration in a scratch-wound assay, with differences in the magnitude of the effect between biomaterials [18]. Taken together, these findings indicate that PRF—including both A-PRF+ and H-PRF variants—can effectively promote periapical healing and dentin bridge formation during direct pulp capping when treating reversible pulpitis. However, standardized application protocols and direct clinical comparisons between distinct PRF formulations are still limited, highlighting the need for further clinical investigations in this field.

The aim of this study is to describe two cases of reversible pulpitis treated by direct pulp capping with PRF membranes (A-PRF+ and H-PRF) and to document the clinical and radiographic outcomes.

## 2. Cases Presentation

Two patients diagnosed with reversible pulpitis were included in this study. In accordance with the Helsinki Declaration II, all participants provided informed written consent. The study protocol was approved by the Research Ethics Committee of the Medical University of Sofia (approval number: 57 from 14/12 July 2024) as part of its framework for scientific research involving human subjects. A single operator treated both of the presented cases. The data is presented on Table 1.

*Patient 1*

*Treating Reversible Pulpitis Through Direct Pulp Capping with A-PRF+*

**Figure 1:**
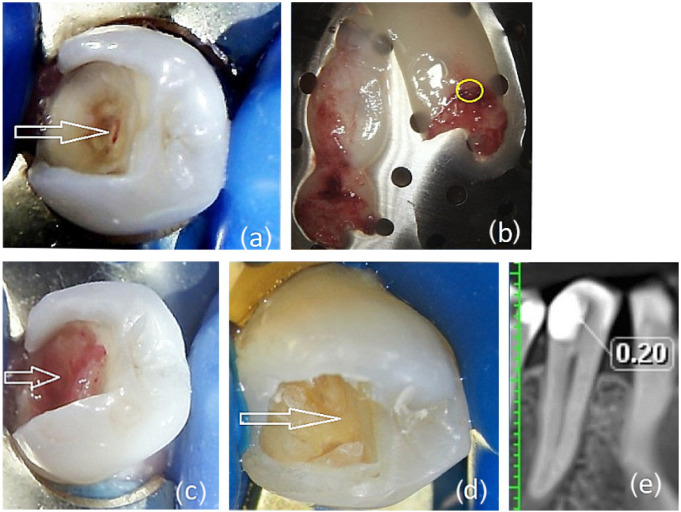
Forty-five-year-old patient with reversible pulpitis treated by direct pulp capping. (**a**) After removing the infected dentin, a pulp exposure communicating with the axial wall was visible (white arrow). (**b**) Prepared A-PRF+ membrane; the yellow circle marks the site within the buffy coat from which the material was harvested. (**c**) The exposure was covered with an A-PRF+ membrane placed on the axial wall of the cavity (white arrow). (**d**) After ninety days, the temporary filling was removed. The white arrow shows no communication. (**e**) CBCT image of tooth 45 after restoration 90 days after beginning treatment. There is no communication, as indicated by the white arrow. The reparative dentin had a thickness of 0.2 mm.

A 45-year-old patient presented with reversible pulpitis on tooth 45. The diagnosis was based on the patient’s medical history (no spontaneous pain), EPT (15 µA, Scorpion, Optica Laser, Sofia, Bulgaria), a radiographic examination, and pulse oximetry (84%, Contec CMS 8000D, Qinhuangdao, China). A deep proximal carious lesion with dark, chronically altered dentin was detected. During caries removal with carbide burs, a small buccal pulp communication (<1.5 mm) was created on the axial wall of the cavity (Figure 1a).

Ten milliliters of venous blood was collected and processed according to Choukroun’s A-PRF+ protocol (DUO Quattro PRF centrifuge, 1300 rpm, 14 min) to obtain an A-PRF+ membrane. The tubes underwent special treatment on their inner walls and were free from anticoagulants. After centrifugation, the tubes were opened for 4–5 min, after which the fibrin clot was separated from the erythrocyte mass without cutting. The harvested material was collected from the buffy coat zone (Figure 1b). Ozone gas was applied to the dentin wound for 18 s to eliminate cariogenic microorganisms within the dentinal wound, after which the pulp exposure and axial wall were covered with the A-PRF+ membrane (Figure 1c). The cavity was temporarily restored with calcium–silicate cement Bio MTA+ (Cerkamed, Stalowa Woda Poland) and resin-modified glass ionomer cement GIC-Fuji LC (CG International Corp., Tokyo, Japan).

After the procedure, the patient reported no pain sensations, emphasizing their comfort. At 90 days, the tooth was asymptomatic, with an EPT value of 9 µA. After removing the temporary restoration, no communication with the pulp was detected (Figure 1d), and a definitive restoration was placed. CBCT examination confirmed intact periapical tissues and complete closure of the pulp communication by reparative dentin, with a thickness of 0.2 mm (Figure 1e).

*Patient 2*

*Treating Reversible Pulpitis Through Direct Pulp Capping with H-PRF*

**Figure 2:**
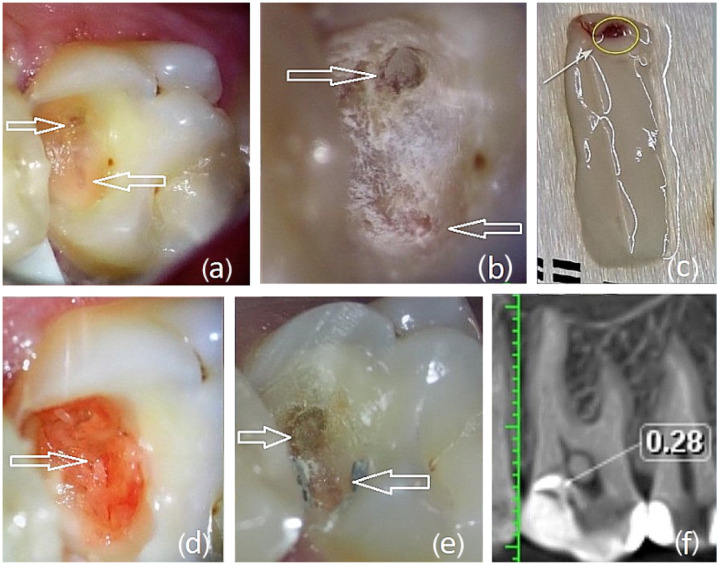
Patient,16 years old, with reversible pulpitis treated by direct pulp capping. (**a**) After removing the infected dentin, the white arrows indicate multiple pulp exposures. (**b**) Following ozonation, the white arrows mark the pulp exposure in dentin; the palatal pulp horn exposure lies below the level of the surrounding dentin. (**c**) The area of the H-PRF membrane used to cover the pulp communication is indicated by a yellow circle. (**d**) The exposure was covered with a H-PRF membrane—the white arrow on the axial wall of the cavity. (**e**) After removing the temporary restoration (day 46), the sites previously marked by white arrows were filled with reparative dentin. (**f**) CBCT (sagittal slice, cropped) of tooth 16 (after day 46) showing no communication with the dental pulp. The reparative dentin had a thickness of 0.28 mm.

A 16-year-old patient presented with reversible pulpitis on tooth 16. The diagnosis was based on the patient’s medical history (no spontaneous pain), EPT (19 µA; Scorpion, Optica Laser, Sofia, Bulgaria), a radiographic examination, and pulse oximetry (85%; Contec CMS 8000D, Qinhuangdao, China). A deep proximal carious lesion with whitish, acutely altered infected dentin was found. After the initial removal of the infected dentin with carbide burs, chemical–mechanical caries removal with Brix 3000 (BRIX S.R.L. Medical Science, Carcarana, Provincia de Santa Fe, Argentina) was performed. Two small pulp exposures (<1.5 mm) were detected on the axial wall of the cavity, buccally and palatally (Figure 2a). The dentin wound was disinfected with ozone gas for 18 s (Figure 2b).

Ten milliliters of venous blood was collected and processed in a Bio-PRF horizontal centrifuge (Bio-PRF, Jupiter, FL, USA) according to Miron’s H-PRF protocol (700 rpm, 8 min) in additive-free tubes with red caps. After centrifugation, the tubes were opened for 4–5 min, after which the fibrin clot was separated from the erythrocyte mass without cutting. The clot was placed on a grid in a Bio-PRF box (Bio-PRF, Jupiter, FL, USA) and compressed into an H-PRF membrane; the buffy coat area was used for pulp capping (Figure 2c). The membrane was adapted over the pulp exposures on the axial wall (Figure 2d), and the cavity was temporarily sealed with Biodentine™ (Septodont, Saint-Maur-des-Fossés, France).

After 46 days, the patient reported mild discomfort to cold. The temporary restoration was removed with the intention of repositioning the H-PRF; however, upon opening the cavity, the palatal depression toward the pulp was completely filled with hard reparative dentin, and no communication was present (Figure 2e). The treatment was completed at the same visit with a glass-ionomer base (Fuji LC, GC International Corp., Tokyo, Japan) and a composite restoration (Diamond, Kulzer, Hanau, Germany). This case demonstrates relatively rapid closure of multiple pulp communications and the formation of a 0.28 mm reparative dentin bridge following direct pulp capping with H-PRF upon CBCT examination (Figure 2f).

The patient was followed up at 6 and 12 months clinically. No complaints were reported, and the values obtained through the EPT test were maintained.

## 3. Discussion

Deep carious lesions harbor a wide range of microorganisms, including oral streptococci, *Gram-positive cocci* (e.g., *Peptostreptococcus* spp.), *Candida*, *Staphylococcus* spp., etc., with oral streptococci predominating and participating in the etiology of dental caries [19]. After removing the infected dentin from the carious lesion, part of the microflora remains on the dentin surface [20]. Therefore, dentin treatment in the presence of pulpal communication is a key factor for the success of direct pulp capping.

Ozone gas has been proposed as an adjuvant for disinfection. Applying ozone to dentin surfaces induces physicochemical changes that support the remineralization and disinfection of hard dental tissues: ozone oxidizes the organic components of demineralized dentin, removes residual bacterial products, facilitates cleansing the dentinal tubules, and reduces the microbial load [20,21]. Its effect is dose- and time-dependent: with short exposures (e.g., 6 s), the reduction in microbial load is incomplete, whereas longer exposures (e.g., 24 s) lead to microorganisms being eradicated [19,20]. At optimal doses, ozone likely acts as a biomodulator of human dental pulp stem cells (DPSCs) [19]. These data were obtained using the Ozone Generator Prozone/OZOTOP device (TIP TOP TIPS, Rolle, Switzerland), which generates ozone gas from atmospheric oxygen at a concentration of approximately 140 ppm and a flow rate of 2 L/min [19,20]. In the clinical cases presented, an exposure time of 18 s (~165 µg) was applied [19].

In the two patients treated, reparative dentin bridge formation was radiographically detectable within different time frames: in the 16-year-old patient, the defect was closed within 46 days, whereas in the 45-year-old patient, closure was observed after 90 days. Age can be regarded as a potential contributing factor, which is in line with the recommendations of the European Society of Endodontology regarding the influence of age on the outcome of vital pulp therapy [22]. In addition, differences in the PRF membranes used in the two cases may also have contributed to the clinical timelines.

PRF is a bioengineered material composed of fibrin and varying amounts of growth factors, platelets, and leukocytes. When applying PRF, the therapeutic aim is not primarily to achieve an antibacterial effect on dentin and pulp (although there is a study confirming the action of H-PRF against *Staphylococcus aureus* and *Escherichia coli* [13]). Rather, owing to their structure and composition, these biomaterials are expected to stimulate the regeneration of the affected dentin and support the restoration of dental pulp function. Leukocytes release signaling molecules that attract stem cells to the injury site and thereby promote tissue regeneration. They act as a cellular reservoir of bioactive mediators and regulate the transition from inflammation to repair by modulating macrophage polarization and extracellular matrix turnover [5,6,23].

In the present report, two different PRF types—A-PRF and H-PRF—were used in the context of direct pulp capping. H-PRF, as described in the literature, contains a higher number of leukocytes (neutrophils, monocytes/macrophages, lymphocytes) embedded in a denser and more highly cross-linked fibrin network compared with certain other PRF formulations [4]. In parallel, H-PRF has been shown to provide a more prolonged release of key growth factors—platelet-derived growth factor (PDGF), transforming growth factor-β1 (TGF-β1), and vascular endothelial growth factor (VEGF)—from both platelets and leukocytes, creating a stable healing microenvironment that may support odontoblast-like differentiation, angiogenesis, and collagen matrix deposition. It has been demonstrated that TGF-β stimulates the proliferation and osteogenic differentiation of DPSCs [24], whereas bone morphogenetic protein-2 (BMP-2) plays a key role in osteoinduction [25]. The actual concentrations of growth factors released from platelets is higher in protocols using horizontal centrifugation. This combination (higher leukocyte content and “slow-release” kinetics of PDGF/TGF-β/VEGF) offers a biologically plausible explanation for the favorable hard-tissue response observed in the case treated with H-PRF. Such kinetics and cellular profiles have been described for leukocyte-rich PRF membranes prepared at lower G-forces or using a horizontal centrifuge and are compatible with the clinical timelines documented in our two cases (≈46 and 90 days) [26,27,28,29,30,31,32]. Nevertheless, the limited number of cases and the differences in clinical context preclude any definitive comparative conclusions.

The favorable outcomes observed in both cases are also in line with the previously demonstrated in vitro migration capacity of human dental pulp cells in response to PRF. In vitro, both A-PRF+ and H-PRF have been reported to increase cell migration compared with untreated control cells (cells cultured under the same conditions without PRF-conditioned medium/extract); H-PRF has been reported to provide a sustained and long-lasting effect, whereas A-PRF+ maintains a moderate stimulating effect over time [18]. These in vitro data support the notion that PRF can act as a biologically active scaffold that enhances cell recruitment and tissue repair.

In both treated cases, we applied a portion of the membrane specifically from the buffy coat zone. It has been shown that A-PRF+ contains a higher number of leukocytes and platelets, particularly in the “buffy coat” fraction, compared with L-PRF [4]. This underlines the importance of membrane positioning and the deliberate use of the most cell-rich portion of the clot when targeting regenerative outcomes.

Filling the defect after closing the pulpal communication in reversible pulpitis with the glass-ionomer cement Fuji LC II is an important step in the treatment process. This material has been shown to possess excellent biocompatibility, comparable to that of calcium hydroxide cements, while at the same time, it provides effective sealing and chemical adhesion to dental tissues [33]. The combination of a biologically active autologous platelet concentrate PRF and a well-sealed GIC/composite restoration may therefore create conditions favorable for the long-term maintenance of pulp vitality.

Overall, these two cases illustrate that direct pulp capping with PRF membranes, combined with ozone-assisted dentin disinfection and adequate coronal sealing, can be associated with maintained pulp vitality and radiographic evidence of reparative dentin bridge formation after carious pulp exposure diagnosed as reversible pulpitis. These observations are consistent with Shobana et al.’s findings regarding the promising potential of PRF membranes in direct pulp capping [16]. The time to clinical closure differed between the two patients; however, this variation cannot be attributed to PRF formulation because the cases were not matched and differed substantially in patient age, lesion characteristics, and clinical context—factors known to influence healing in vital pulp therapy. Therefore, the present report should be interpreted as descriptive and hypothesis-generating rather than comparative. Prospective controlled clinical studies with standardized diagnostic criteria, hemostasis protocols, and validated outcome measures are required to clarify whether specific PRF formulations or preparation protocols influence the timing and quality of reparative dentin formation in direct pulp capping.

## 4. Conclusions

This report describes two cases of reversible pulpitis treated by direct pulp capping with PRF membranes (A-PRF+ and H-PRF) obtained using vertical and horizontal centrifugation, respectively. Both cases showed favorable clinical evolution with maintained vitality and radiographic evidence of reparative dentin bridge formation following carious pulp exposure and definitive coronal sealing. Because the present report includes only two non-matched cases with major confounding factors (notably age and lesion characteristics), no comparative conclusions can be drawn regarding the relative effectiveness of A-PRF+ vs. H-PRF. Larger prospective controlled studies with standardized protocols and validated outcome assessments are needed.

## Figures and Tables

**Table 1:** Clinical evaluation for the presented cases.

	Patient 1	Patient 2
Tooth	45	16
Age of the patient	45	16
Sex	female	male
EPT	15 µA	19 µA
Pulse oximetry	84%	85%
Spontaneous pain	No	No
Size of the caries lesion	Deep proximal caries distally	Deep proximal caries distally
Pulp exposure (mm) with measuring probe	Around < 1.5 mm	Around < 1.5 mm
Shape of pulp communication	oval	irregular
Hemostasis time after pulp exposure	1–2 s.	1–2 s.
Diagnosis	Reversible pulpitis	Reversible pulpitis
Disinfection	Ozone, 18 s	Ozone, 18 s
Type of autologous platelet concentrates	A-PRF+	H-PRF
Type of centrifuge and regime	Vertical 1300 rpm 14 min	Horizontal 700 rpm 8 min
Collection site from autologous platelet concentrates	Buffy coat zone	
Restorative material	Bio-MTA+ Glass-ionomer cement	Biodentin Glass-ionomer cement
Time to clinical closure (days)	After 90 days	After 46 days

## Data Availability

The data presented in this study are available from the corresponding author upon request.

## Abbreviations

A-PRF+	advanced platelet-rich fibrin plus
H-PRF	horizontal platelet-rich fibrin
PRF	platelet-rich fibrin
CBCT	cone-beam computed tomography

## References

[1] Quirynen M., Blanco J., Wang H.L., Donos N., Temmerman A., Castro A., Pinto N. (2025). Instructions for the use of L-PRF in different clinical indications. Periodontology 2000.

[2] Upputuri P.K., Sivasubramanian K., Mark C.S., Pramanik M. (2015). Recent developments in vascular imaging techniques in tissue engineering and regenerative medicine. BioMed Res. Int..

[3] Ghanaati S., Al-Maawi S., Herrera-Vizcaino C., Alves G.G., Calasans-Maia M.D., Sader R., Kirkpatrick C.J., Choukroun J., Bönig H., Mourão C.F.A.B. (2018). A proof of the low-speed centrifugation concept in rodents: New perspectives for in vivo research. Tissue Eng. Part C Methods.

[4] Farshidfar N., Amiri M.A., Estrin N.E., Ahmad P., Sculean A., Zhang Y., Miron R.J. (2025). Platelet-rich plasma (PRP) versus injectable platelet-rich fibrin (i-PRF): A systematic review across all fields of medicine. Periodontology 2000.

[5] Quirynen M., Siawash S.A.M., Yu J., Miron R.J. (2025). Essential principles for blood centrifugation. Periodontology 2000.

[6] Fujioka-Kobayashi M., Miron R.J., Hernandez M., Kandalam U., Zhang Y., Choukroun J. (2017). Optimized Platelet-Rich Fibrin With the Low-Speed Concept: Growth Factor Release, Biocompatibility, and Cellular Response. J. Periodontol..

[7] Fujioka-Kobayashi M., Kono M., Katagiri H., Schaller B., Zhang Y., Sculean A., Miron R.J. (2021). Histological comparison of platelet-rich fibrin clots prepared by fixed-angle versus horizontal centrifugation. Platelets.

[8] Ponte A.L., Marais S., Gallay N., Langonné A., Delorme B., Hérault O., Charbord P., Domenech J. (2007). The in vitro migration capacity of human bone marrow mesenchymal stem cells: Comparison of chemokine and growth factor chemotactic activities. Stem Cells.

[9] Farshidfar N., Apaza Alccayhuaman K.A., Estrin N.E., Ahmad P., Sculean A., Zhang Y., Miron R.J. (2025). Advantages of horizontal centrifugation of platelet-rich fibrin in regenerative medicine and dentistry. Periodontology 2000.

[10] Miron R.J., Moraschini V., Estrin N., Shibli J.A., Cosgarea R., Jepsen K., Jervøe-Storm P.M., Wang H.-L., Sculean A., Jepsen S. (2025). Autogenous platelet concentrates for treatment of intrabony defects—A systematic review with meta-analysis. Periodontology 2000.

[11] Miron R.J., Chai J., Zheng S., Feng M., Sculean A., Zhang Y. (2019). A novel method for evaluating and quantifying cell types in platelet-rich fibrin and an introduction to horizontal centrifugation. J. Biomed. Mater. Res. A.

[12] Miron R.J., Fujioka-Kobayashi M., Sculean A., Zhang Y. (2024). Optimization of platelet-rich fibrin. Periodontology 2000.

[13] Feng M., Wang Y., Zhang P., Zhao Q., Yu S., Shen K., Miron R.J., Zhang Y. (2020). Antibacterial effects of platelet-rich fibrin produced by horizontal centrifugation. Int. J. Oral Sci..

[14] Kirilova J., Kirov D., Yovchev D., Topalova-Pirinska S., Deliverska E. (2022). Endodontic and surgical treatment of chronic apical periodontitis: A randomized clinical study. Biotechnol. Biotechnol. Equip..

[15] Darwish O.B., Aziz S.M.A., Sadek H.S. (2025). Healing potentiality of blood clot, S-PRF and A-PRF as scaffold in treatment of non-vital mature single-rooted teeth with chronic periapical periodontitis following regenerative endodontic therapy: Randomized clinical trial. BMC Oral Health.

[16] Shobana S., Kavitha M., Srinivasan N. (2022). Efficacy of platelet-rich plasma and platelet-rich fibrin for direct pulp capping in adult patients with carious pulp exposure—A randomised controlled trial. Eur. Endod. J..

[17] Kirilova J.N., Kirilova Kosturkov D. (2023). Direct pulp capping with advanced platelet-rich fibrin: A report of two cases. Medicina.

[18] Kirilova J.N., Vladova R.Z., Petrova V.P., Yantcheva S., Deliverska E.G., Ishkitiev N.D. (2025). Influence of different biomaterials extracted from autologous blood on the cell migration of stem cells from dental pulp. J. Funct. Biomater..

[19] Kirilova J. (2013). Glass-Ionomer Cements as a Biomaterial for the Treatment of Proximal Caries in Posterior Teeth (“Sandwich” Restorations). Ph.D. Thesis.

[20] Kirilova J., Topalova-Pirinska S., Kirov D., Deliverska E., Doichinova L. (2019). Types of microorganisms in proximal caries lesion and ozone treatment. Biotechnol. Biotechnol. Equip..

[21] Pasalkar L., Chavan M., Kharat A., Sanap A., Kheur S., Bhonde R. (2022). Gaseous ozone treatment augments chondrogenic and osteogenic differentiation but impairs adipogenic differentiation in human dental pulp stem cells in vitro. J. Orofac. Sci..

[22] Duncan H.F., Galler K.M., Tomson P.L., Simon S., El-Karim I., Kundzina R., Krastl G., Dammaschke T., Fransson H., Markvart M. (2019). European Society of Endodontology position statement: Management of deep caries and the exposed pulp. Int. Endod. J..

[23] Lee Y.C., Chan Y.H., Hsieh S.C., Lew W.Z., Feng S.W. (2019). Comparing the Osteogenic Potentials and Bone Regeneration Capacities of Bone Marrow and Dental Pulp Mesenchymal Stem Cells in a Rabbit Calvarial Bone Defect Model. Int. J. Mol. Sci..

[24] Gao P., Liu C., Dong H., Li Q., Chen Y. (2023). TGF-β promotes the proliferation and osteogenic differentiation of dental pulp stem cells: A systematic review and meta-analysis. Eur. J. Med. Res..

[25] Mazzucchi G., Mariano A., Serafini G., Lamazza L., Scotto d’Abusco A., De Biase A., Lollobrigida M. (2024). Osteoinductive properties of autologous dentin: An ex vivo study on extracted teeth. J. Funct. Biomater..

[26] Kobayashi E., Flückiger L., Fujioka-Kobayashi M., Sawada K., Sculean A., Schaller B., Miron R.J. (2016). Comparative release of growth factors from PRP, PRF, and advanced-PRF. Clin. Oral Investig..

[27] Miron R.J., Fujioka-Kobayashi M., Hernandez M., Kandalam U., Zhang Y., Ghanaati S., Choukroun J. (2017). Injectable platelet-rich fibrin (i-PRF): Opportunities in regenerative dentistry?. Clin. Oral Investig..

[28] Fujioka-Kobayashi M., Abd El Raouf M., Saulacic N., Kobayashi E., Zhang Y., Schaller B., Miron R.J. (2019). Superior bone-inducing potential of rhBMP9 compared to rhBMP2. J. Biomed. Mater. Res. A.

[29] Dohan Ehrenfest D.M., Pinto N.R., Pereda A., Jiménez P., Del Corso M., Kang B.S., Nally M., Lanata N., Wang H.-L., Quirynen M. (2018). The impact of the centrifuge characteristics and centrifugation protocols on the cells, growth factors, and fibrin architecture of an L-PRF clot and membrane. Platelets.

[30] Zwittnig K., Kirnbauer B., Jakse N., Schlenke P., Mischak I., Ghanaati S., Al-Maawi S., Végh D., Payer M., Zrnc T.A. (2022). Growth factor release within liquid and solid PRF. J. Clin. Med..

[31] Miron R.J., Bohner M., Zhang Y., Bosshardt D.D. (2024). Osteoinduction and osteoimmunology: Emerging concepts. Periodontology 2000.

[32] Galarraga-Vinueza M.E., Barootchi S., Nevins M.L., Nevins M., Miron R.J., Tavelli L. (2024). Twenty-five years of recombinant human growth factors rhPDGF-BB and rhBMP-2 in oral hard and soft tissue regeneration. Periodontology 2000.

[33] Kirilova J. (2024). A study of the cytotoxicity of resin-modified glass-ionomer cements. MedInform.

